# Tumor Necrosis Factor α Inhibits Expression of the Iron Regulating Hormone Hepcidin in Murine Models of Innate Colitis

**DOI:** 10.1371/journal.pone.0038136

**Published:** 2012-05-31

**Authors:** Nanda Kumar N. Shanmugam, Shiri Ellenbogen, Estela Trebicka, Lijian Wang, Subhankar Mukhopadhyay, Adam Lacy-Hulbert, Carey Ann Gallini, Wendy S. Garrett, Bobby J. Cherayil

**Affiliations:** 1 Mucosal Immunology Laboratory, Massachusetts General Hospital, Boston, Massachusetts, United States of America; 2 Developmental Immunology Laboratory, Department of Pediatrics, Massachusetts General Hospital, Boston, Massachusetts, United States of America; 3 Sir William Dunn School of Pathology, University of Oxford, Oxford, United Kingdom; 4 Harvard Medical School, Boston, Massachusetts, United States of America; 5 Departments of Immunology and Infectious Diseases and Genetics and Complex Diseases, Harvard School of Public Health, Boston, Massachusetts, United States of America; 6 Dana-Farber Cancer Institute, Harvard School of Public Health, Boston, Massachusetts, United States of America; Juntendo University School of Medicine, Japan

## Abstract

**Background:**

Abnormal expression of the liver peptide hormone hepcidin, a key regulator of iron homeostasis, contributes to the pathogenesis of anemia in conditions such as inflammatory bowel disease (IBD). Since little is known about the mechanisms that control hepcidin expression during states of intestinal inflammation, we sought to shed light on this issue using mouse models.

**Methodology/Principal Findings:**

Hepcidin expression was evaluated in two types of intestinal inflammation caused by innate immune activation—dextran sulfate sodium (DSS)-induced colitis in wild-type mice and the spontaneous colitis occurring in T-bet/Rag2-deficient (TRUC) mice. The role of tumor necrosis factor (TNF) α was investigated by in vivo neutralization, and by treatment of a hepatocyte cell line, as well as mice, with the recombinant cytokine. Expression and activation of Smad1, a positive regulator of hepcidin transcription, were assessed during colitis and following administration or neutralization of TNFα. Hepcidin expression progressively decreased with time during DSS colitis, correlating with changes in systemic iron distribution. TNFα inhibited hepcidin expression in cultured hepatocytes and non-colitic mice, while TNFα neutralization during DSS colitis increased it. Similar results were obtained in TRUC mice. These effects involved a TNFα-dependent decrease in Smad1 protein but not mRNA.

**Conclusions/Significance:**

TNFα inhibits hepcidin expression in two distinct types of innate colitis, with down-regulation of Smad1 protein playing an important role in this process. This inhibitory effect of TNFα may be superseded by other factors in the context of T cell-mediated colitis given that in the latter form of intestinal inflammation hepcidin is usually up-regulated.

## Introduction

Inflammatory conditions are often accompanied by a debilitating anemia known as the anemia of inflammation (AI) [1]. The pathogenesis of AI is related to abnormally elevated levels of the liver peptide hormone hepcidin, a key regulator of systemic iron metabolism [2]. Hepcidin binds to and down-regulates the iron exporter ferroportin, which is expressed on macrophages and duodenal enterocytes, thereby inhibiting, respectively, the recycling of iron from erythrocytes and the absorption of iron from the diet [3]. Thus, elevated circulating hepcidin leads to decreased serum iron, consequent iron-restricted impairment of erythropoiesis and, ultimately, anemia.

Anemia is a common feature of IBD and is multi-factorial in origin [4]. With the discovery of the role played by hepcidin in AI, recent investigations have attempted to determine whether this hormone is involved in the anemia of IBD. The results have not always been consistent. Urinary hepcidin was found to be significantly elevated in a study of children with active Crohn's disease, correlating with decreased iron absorption from the gut [5]. An investigation of adults with IBD also demonstrated elevated serum hepcidin levels in the patients that correlated positively with disease activity and negatively with hemoglobin [6]. On the other hand, an analysis of adults with both active and inactive IBD revealed lower serum hepcidin in patients compared to controls [7]. The reason for the discordance between the different clinical studies is not clear. Hepcidin expression has also been examined in mouse models of colitis. We found that liver hepcidin expression was up-regulated in the piroxicam/IL-10 knockout colitis model and the Rag knockout/T cell transfer colitis model [8], [9], while other investigators have demonstrated elevated hepcidin levels in the spontaneous ilietis of TNFΔARE mice [10]. Moreover, we showed that reducing hepcidin expression in the T cell transfer colitis model led to a significant improvement of serum iron levels, consistent with the idea that elevated hepcidin contributes to the pathogenesis of the anemia associated with intestinal inflammation [9].

Hepcidin expression in the liver is regulated exclusively at the transcriptional level, and is determined by the integration of multiple signaling inputs acting on several key transcription factors [11]. Serum iron is a major influence, and is sensed via 2 hepatocyte surface proteins, the hereditary hemochromatosis protein HFE and the type 2 transferrin receptor, that interact to activate signals leading to increased hepcidin expression [12]–[14]. Inflammation is an additional stimulus that up-regulates hepcidin. IL-6 is an important mediator of this response following injection of lipopolysaccharide or turpentine, and functions by activating the transcription factor STAT3 [15]–[18]. The induction of hepcidin in response to either inflammatory stimuli or elevated serum iron is dependent on signals provided by a sub-set of bone morphogenetic proteins (BMPs). Several BMPs, including BMPs 2, 4 and 6, up-regulate hepcidin in tissue culture systems [19], but BMP6 is probably the major regulator of hepcidin expression in vivo [20], [21]. Deficiency of BMP6 in mice results in low basal hepcidin expression, progressive systemic iron overload, and significantly compromised induction of hepcidin in response to lipopolysaccharide administration [20], [21]. Further supporting the important role played by BMP-activated signals in controlling hepcidin expression, we found that neutralization of BMP6, either alone or together with BMPs 2 and 4, significantly inhibited hepcidin up-regulation during T cell transfer colitis [9]. Signaling by BMPs 2, 4 and 6 involves specific type I and type II receptors and the co-receptor hemojuvelin, and leads to the phosphorylation of the receptor-associated Smad proteins (Smads 1, 5 and 8), which then associate with the transcription factor Smad4 to induce hepcidin expression [22]. Counteracting the signals that increase hepcidin expression, anemia and hypoxia, as well as decreases in circulating iron and body iron stores, lead to the down-regulation of hepcidin expression [11].

The mechanisms that regulate hepcidin expression are complex. How these mechanisms operate in the context of clinically relevant inflammation, such as that occurring in IBD, is not well understood. Shedding light on this issue is important not only for our basic understanding of how hepcidin expression is controlled in vivo, but also in terms of clarifying the pathogenesis of IBD-associated anemia and devising ways to treat this condition. Accordingly, we carried out experiments to determine how hepcidin expression is regulated in mouse models of IBD.

## Materials and Methods

### Ethics statement

All animal studies were carried out in accordance with the recommendations in the Guide for the Care and Use of Laboratory Animals of the National Institutes of Health. The protocol was approved by the Sub-committee on Research Animal Care of Massachusetts General Hospital (protocol number 2008N000061, animal welfare assurance number A3596-01).

### Animal studies

Wild-type, 8 week old female C57BL/6 mice were purchased from the Jackson Laboratory and were given 3% DSS in their drinking water for 3 or 7 days. Untreated controls received regular drinking water. TRUC mice (BALB/c background) and the characteristics of their intestinal inflammation, have been described previously [23]. Female TRUC mice and Rag2 knockout controls were used starting at 4 weeks of age. All mice were maintained under specific pathogen free conditions.

### Analysis of hepatic gene expression

After mice were euthanized, pieces of liver were homogenized in TRIzol reagent (Life Technologies, Grand Island, NY). Total RNA was prepared and used to determine hepcidin and Smad1 expression by quantitative reverse transcriptase-polymerase chain reaction (qRT-PCR), essentially as described previously [8]. Relative expression was calculated using the 2^−ΔΔCt^ method after normalizing to a housekeeping transcript, GAPDH or actin. Primers used to amplify hepcidin, Id1, GAPDH and actin have been published earlier [8], [9], [21]. Primers for amplification of Smad1 were: forward, 5′GACGCTTTGGTGAAGAAACTGA3′, reverse, 5′ACACGGCAATAAATGACATGAG3′.

### Serum transaminase estimation

Serum collected from mice at the time of sacrifice was analyzed for transaminase levels by the Veterinary Clinical Pathology Laboratory at Massachusetts General Hospital.

### Estimation of serum and tissue iron levels

Serum iron was estimated as described previously using a colorimetric assay kit from Thermo Scientific [9]. Splenic iron was measured as described previously [19].

### Immunoblotting

Liver tissue was homogenized in lysis buffer containing protease and phosphatase inhibitors as described earlier [8], [9]. After gel electrophoresis and transfer to nitrocellulose membranes, the lysates were blotted with antibodies to the phosphorylated form of Smad1/5/8, total Smad1 (Cell Signaling Technology), total Smad 1/5/8 (Abcam), actin or GAPDH (Sigma). The blots were developed with fluorochrome-conjugated secondary antibodies and visualized using the Odyssey infra-red fluorescence imaging system (LI-COR Biosciences, Lincoln, NE). Band fluorescence intensities were quantified after background subtraction and used to calculate the changes in relative amounts of the corresponding proteins.

### Assessment of intestinal inflammation

The colon was excised, its length measured and segments were placed in tissue culture medium overnight. The culture supernatants were then used to estimate the levels of various cytokines by ELISA, as previously described [8]. The cytokine concentrations were normalized to the total protein concentration of a homogenate of the corresponding colon segment and expressed as ng/mg. In some experiments, colon pro-inflammatory cytokine expression was assessed by qRT-PCR as described previously [8], [9].

### Tissue culture studies

Huh7 human hepatoma cells [24] were obtained from Dr. Raymond Chung, Massachusetts General Hospital. The cells were seeded in 12-well tissue culture plates and treated with various concentrations of recombinant human TNFα or IL-6 (R&D Systems) for different time periods. Total RNA was prepared using TRIzol and used to estimate hepcidin expression by qRT-PCR. Primers used to amplify human hepcidin were: forward, 5′CTCTGTTTTCCCACAACAGAC3′, reverse, 5′TAGGGGAAGTGGGTGTCTC3′. Relative expression was calculated using the 2^−ΔΔCt^ method after normalizing to actin. In some experiments, the cells were transiently transfected with a firefly luciferase reporter driven by the human hepcidin promoter [25] using Lipofectamine 2000 (Life Technologies, Grand Island, NY), along with a constitutively expressed Renilla luciferase reporter. Forty-eight hours after transfection, the cells were treated with recombinant TNFα. Cell lysates were prepared and used to estimate firefly luciferase activity normalized to Renilla luciferase using a Dual-Luciferase kit (Promega, Madison, WI).

### In vivo manipulation of TNFα

Non-colitic wild-type mice were injected intraperitoneally with recombinant TNFα (R&D Systems), either 25 µg/kg body weight per dose given at time 0 and 2 hours followed by sacrifice at 6 hours, or 50 µg/kg body weight given at time 0 followed by sacrifice at 16 hours. Control mice were injected with equivalent volumes of PBS according to the same schedule. Based on earlier studies of TNFα neutralization in mice [26], we administered the anti-TNFα antibody infliximab (Remicade, Centocor Ortho Biotech, Malvern, PA) by intraperitoneal injection at 10 mg/kg body weight per dose. In the DSS colitis model, the antibody was injected on alternate days starting on the day DSS was initiated, resulting in a total of 4 doses by the end of 7 days. Control animals were injected with an equivalent volume of PBS at the same times that the anti-TNFα treated mice received the antibody injections. TNFα neutralization in TRUC mice was carried out as described previously [23], starting at 4 weeks of age (hamster anti-mouse TNFα antibody IgG1 clone TN3-19.12 or an isotype-matched control antibody, both obtained from Bio Express Inc., intraperitoneally injected at 15 mg/kg once weekly for 4 weeks). In all neutralization experiments, the animals were sacrificed one day after the last dose of antibody.

### Statistical analysis

The student's t test was used to compare results between groups. A p value of <0.05 was considered to be statistically significant. In the figures, statistically significant differences are indicated with an asterisk. Where the difference is close to significance, the actual p value is noted.

## Results

**Figure 1:**
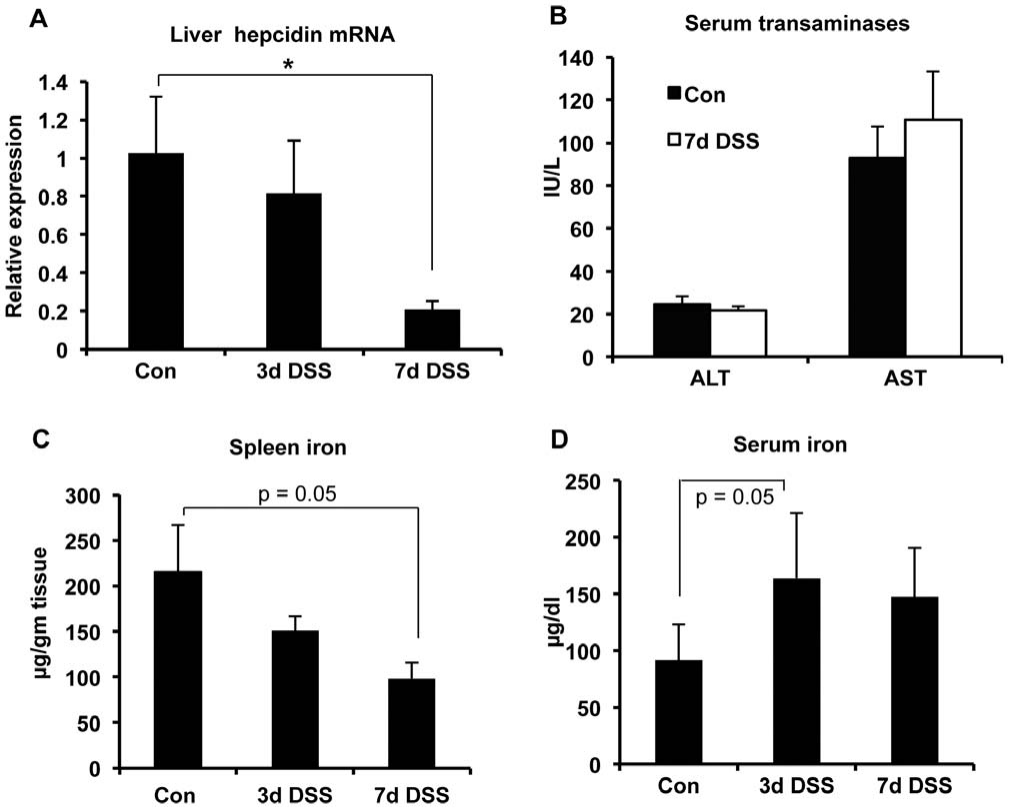
Alterations of iron metabolism in DSS colitis. **A,** Hepcidin mRNA levels, normalized to GAPDH, in livers of control, untreated mice (Con), or mice treated for 3 or 7 days with DSS. Expression is indicated relative to the mean of the control animals. *p = 0.01, n = 7 mice per group. **B,** Serum transaminase levels in control, untreated mice (Con), and mice treated for 7 days with DSS. n = 10 for the control group and 8 in the DSS treated group. **C,** Splenic iron concentrations in control, untreated mice (Con), or mice treated for 3 or 7 days with DSS. n = 7 in each group. **D,** Serum iron concentrations in control, untreated mice (Con), or mice treated for 3 or 7 days with DSS. n = 4 (control), 5 (3d DSS), 4 (7d DSS).

We showed earlier that liver hepcidin expression increased during the initial 48 hours of DSS treatment, and that the up-regulation was dependent on IL-6 [9]. When we extended the time of DSS treatment, we were surprised to find that hepcidin expression (as determined by qRT-PCR) was significantly reduced by 7 days (Figure 1A) and remained at a persistently low level for as long as 2 weeks (not shown). The reduction of hepcidin expression was unlikely to be the result of a generalized impairment of liver function because serum transaminase levels did not change significantly (Figure 1B). Since an assay to measure levels of the hepcidin peptide is not generally available, we determined if the changes in hepcidin mRNA were associated with alterations in tissue iron distribution. A decrease in hepcidin is expected to lead to increased ferroportin expression on phagocytes of the spleen and a consequent efflux of iron from these cells into the circulation [3]. Accordingly, we measured serum and splenic iron concentrations. In keeping with the down-regulation of hepcidin, we found clear trends (p = 0.05) towards decreased splenic iron (Figure 1C) and increased serum iron (Figure 1D) in the mice with DSS colitis.

**Figure 2:**
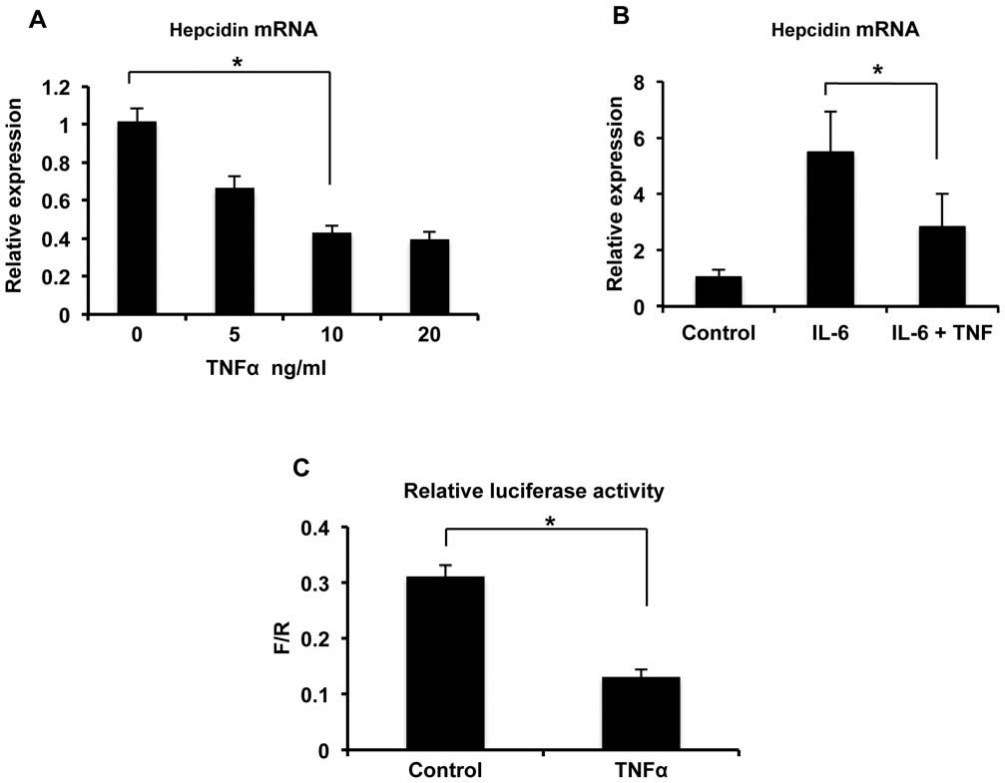
Effects of TNFα on hepcidin expression in Huh7 cells. **A**, Huh7 cells were treated with the indicated concentrations of TNFα for 12 hours and hepcidin expression, normalized to actin, was determined relative to the mean of untreated cells. *p<0.0001, n = 5 for each condition. **B**, Huh7 cells were treated for 12 hours with 10 ng/ml of IL-6, either alone or together with 10 ng/ml of TNFα, and hepcidin expression, normalized to actin, was determined relative to the mean of control, untreated cells. *p = 0.014, n = 3 for each condition. **C**, Huh7 cells were transfected with a firefly luciferase reporter under the control of the hepcidin promoter along with a constitutively expressed Renilla reporter. Forty-eight hours after transfection, the cells were treated with 10 ng/ml of TNFα for 12 hours and luciferase activities determined. The ratio of firefly to Renilla (F/R) luciferase activity is indicated. *p = 0.002, n = 3 for each condition.

To investigate the mechanism of DSS colitis-induced down-regulation of hepcidin, we considered the possibility that TNFα might be involved, since expression of this cytokine is elevated in the colon (Figure S1A) and liver (data not shown) as part of the inflammatory process. We tested this idea initially in the Huh7 hepatoma cell line, where we were able to demonstrate a dose-dependent inhibition of hepcidin expression following treatment with TNFα (Figure 2A). Furthermore, TNFα was able to inhibit the up-regulation of hepcidin induced by IL-6 in the Huh7 cells (Figure 2B), which could help to explain why hepcidin expression was decreased during DSS colitis despite increased levels of IL-6 produced by the colon (Figure S1B). The effect of TNFα was at the level of transcription, as indicated by transient transfection of Huh7 cells with a luciferase reporter driven by the hepcidin promoter (Figure 2C). The TNFα-induced down-regulation of hepcidin in Huh7 cells was not the result of cytotoxicity as indicated by a lactate dehydrogenase release assay (Figure S2).

**Figure 3:**
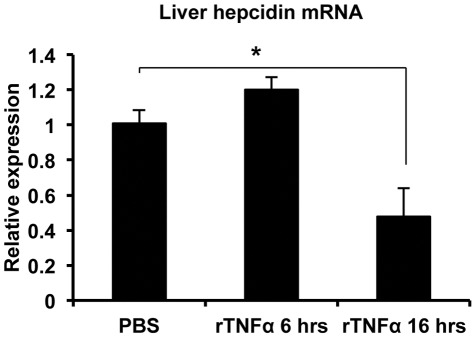
Effect of TNFα on hepcidin expression in vivo. Mice without colitis were treated with PBS or recombinant TNFα (rTNFα) for 6 or 16 hours as described in the text. Liver hepcidin expression was analyzed and is shown relative to the mean of PBS-treated mice after normalizing to GAPDH. *p = 0.005, n = 8 (PBS), 6 (each of the TNFα treated groups).

**Figure 4:**
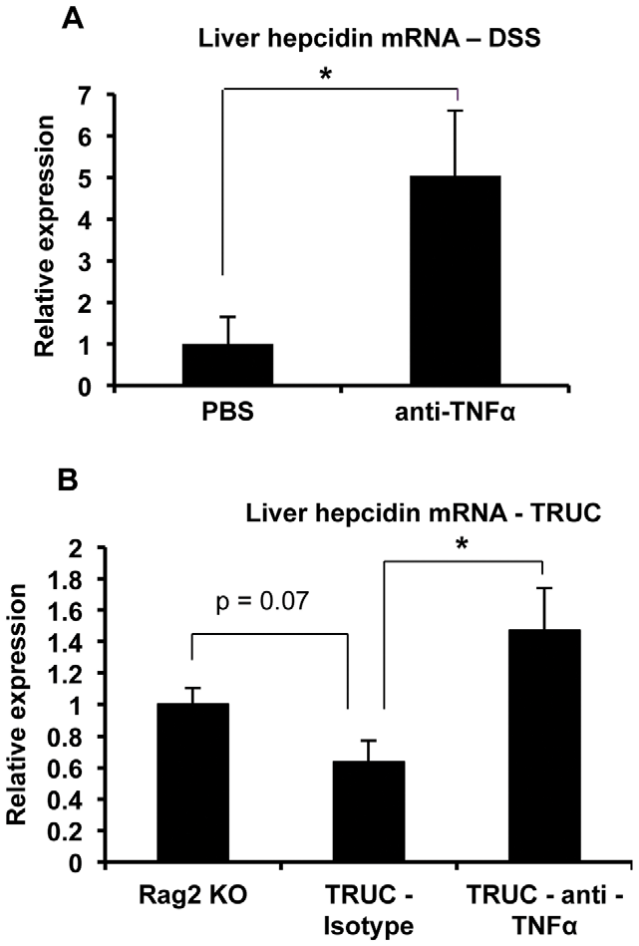
Effect of TNFα neutralization on hepcidin expression in DSS colitis. **A**, Mice with DSS-induced colitis were treated with neutralizing anti-TNFα antibody or vehicle (PBS) as described in the text. Liver hepcidin expression was analyzed and is shown relative to the mean of PBS-treated mice after normalizing to GAPDH. *p = 0.03, n = 5 (PBS), 10 (anti-TNFα). **B**, TRUC mice were treated with a neutralizing anti-TNFα antibody or an isotype-matched control antibody as described in the text. Liver hepcidin levels were measured and is shown relative to the mean of Rag2 knockout (KO) mice without colitis after normalizing to GAPDH. *p = 0.02, n = 4 (Rag2 KO), 5 (TRUC isotype), 5 (TRUC anti-TNFα).

To assess the role of TNFα in hepcidin regulation in vivo, we injected wild-type mice without colitis with the recombinant cytokine and found, consistent with the results in the Huh7 cells, that hepcidin expression was significantly reduced (Figure 3). Serum transaminase estimation did not indicate any hepatoxicity resulting from the TNFα treatment (data not shown). We then tested the effect of the TNFα neutralizing antibody infliximab in mice with DSS colitis. The efficacy of neutralization was confirmed by our finding that the severity of intestinal inflammation, as indicated by colon length and colon IL-6 levels, was significantly reduced in the animals treated with infliximab (Figure S3). In addition, hepcidin expression was significantly elevated in the infliximab-treated colitic mice (Figure 4A). In aggregate, our results indicate that hepcidin expression is inhibited during DSS colitis by a TNFα-dependent mechanism.

We wished to determine whether TNFα-mediated inhibitionof hepcidin expression was a unique feature of DSS colitis. Accordingly, we evaluated another model of intestinal inflammation caused by innate immune activation – spontaneous colitis occurring in TRUC mice, which have a combined deficiency of Rag2 and T-bet [23]. The lack of T-bet and lymphocytes in these animals results in excessive TNFα production by colonic dendritic cells, with consequent enterocyte apoptosis, colonic barrier disruption, alterations in the microbiota, and development of a progressive, TNFα-dependent colitis starting at about 4 weeks of age. Cohorts of 4 week old TRUC mice were treated for 4 weeks with weekly injections of a neutralizing hamster anti-mouse TNFα antibody [23] or an isotype-matched control. Compared to similarly aged, non-colitic Rag2 knockout mice, the isotype-treated TRUC mice with colitis displayed a trend (p = 0.07) towards lower hepcidin levels, while anti-TNFα treatment of the TRUC mice led to a significant increase in hepcidin (Figure 4B). Thus, our findings demonstrate that TNFα has an inhibitory effect on hepcidin expression in a second model of innate colitis.

**Figure 5:**
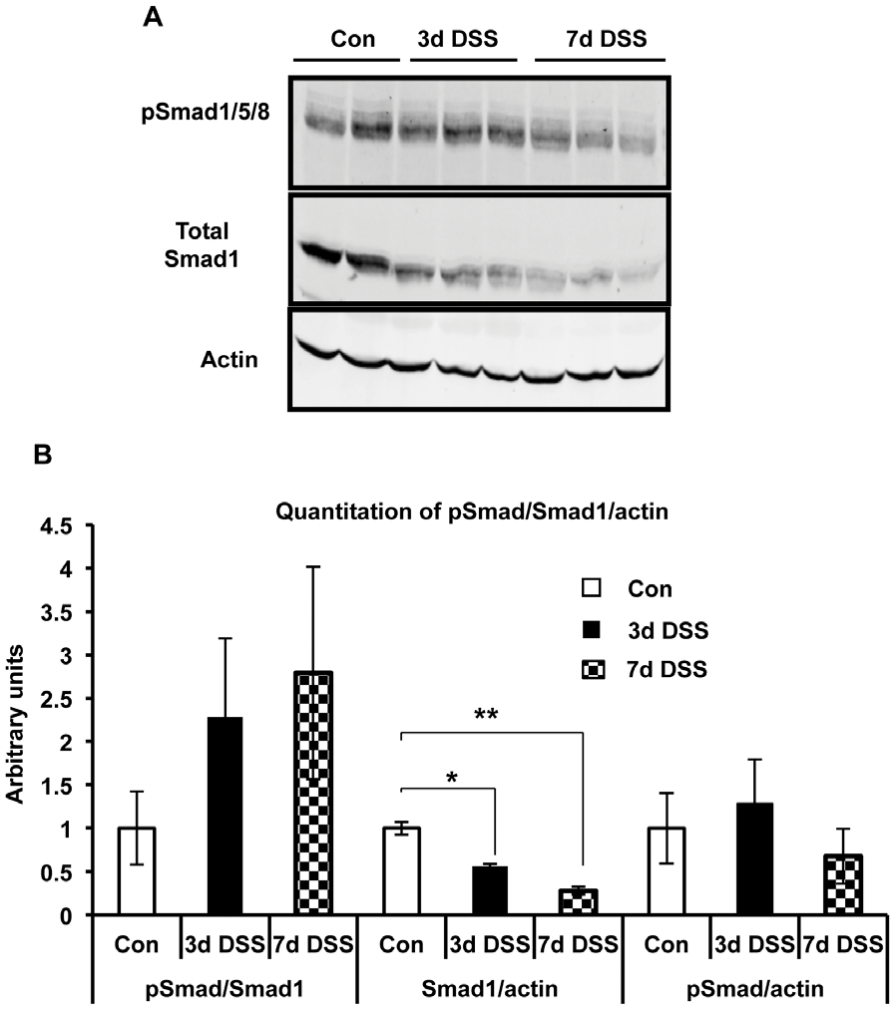
Alterations in the BMP/Smad pathway in DSS colitis. **A**, Representative immunoblotting of liver lysates for phosphorylated Smad (pSmad) 1/5/8, total Smad1 and actin in control untreated mice (Con), or mice treated for 3 or 7 days with DSS. Each lane represents an individual animal. **B**, Quantitation of band intensities from the immunoblotting experiment. *p = 0.001, **p = 0.001, n = 6 mice per group.

**Figure 6:**
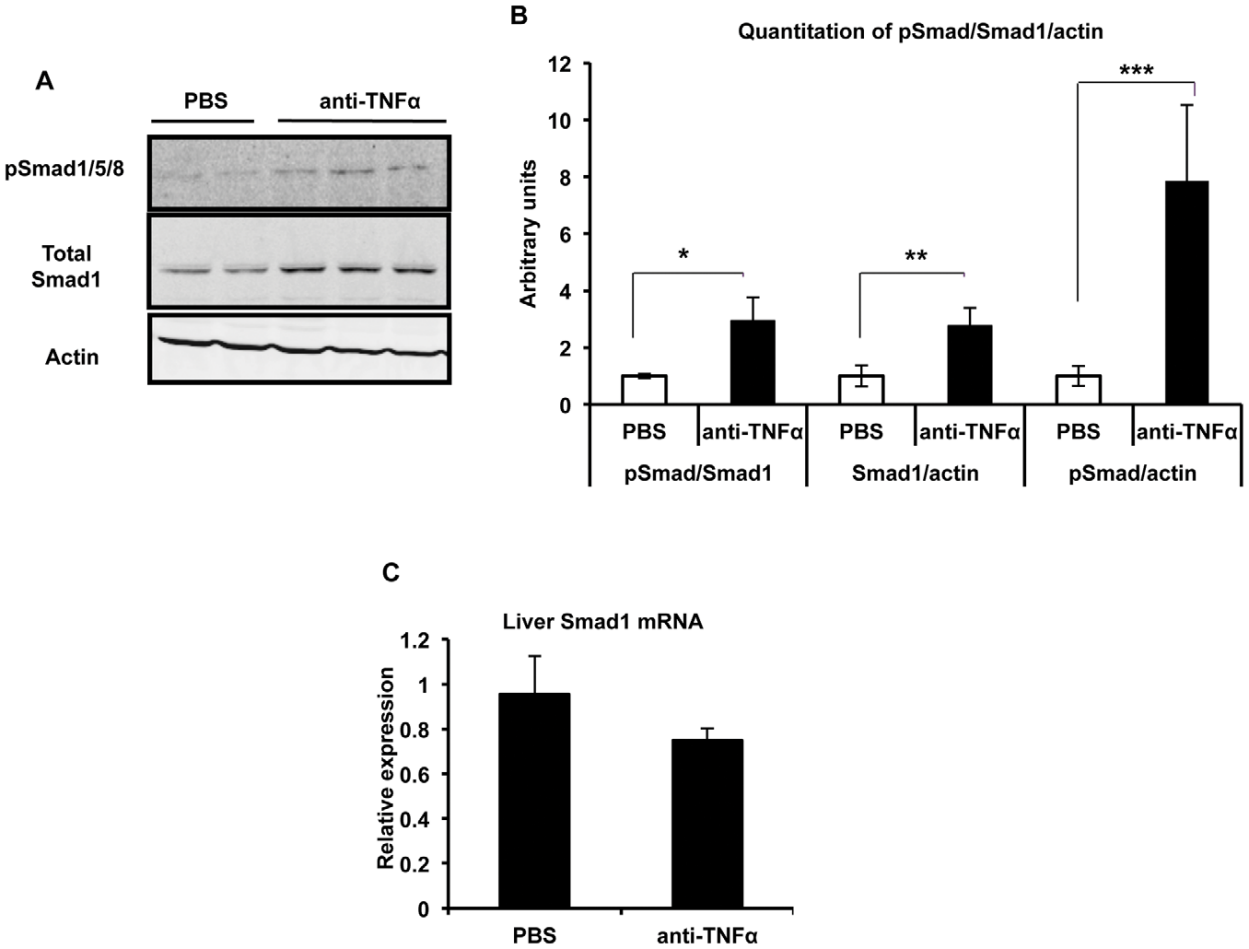
Effects of TNFα neutralization on the BMP/Smad pathway in DSS colitis. **A**, Representative immunoblotting of liver lysates for phosphorylated Smad (pSmad) 1/5/8, total Smad1 and actin in mice with DSS colitis that were treated with anti-TNFα or vehicle (PBS) for 7 days. Each lane represents an individual animal. **B**, Quantitation of band intensities from immunoblotting experiment. *p = 0.05, **p = 0.02, ***p = 0.02, n = 5 (PBS), 10 (anti-TNFα). **C**, Smad1 mRNA expression in mice with DSS colitis that were treated with anti-TNFα or vehicle (PBS) for 7 days, shown relative to the mean of PBS-treated mice after normalizing to GAPDH. n = 5 (PBS), 10 (anti-TNFα).

**Figure 7:**
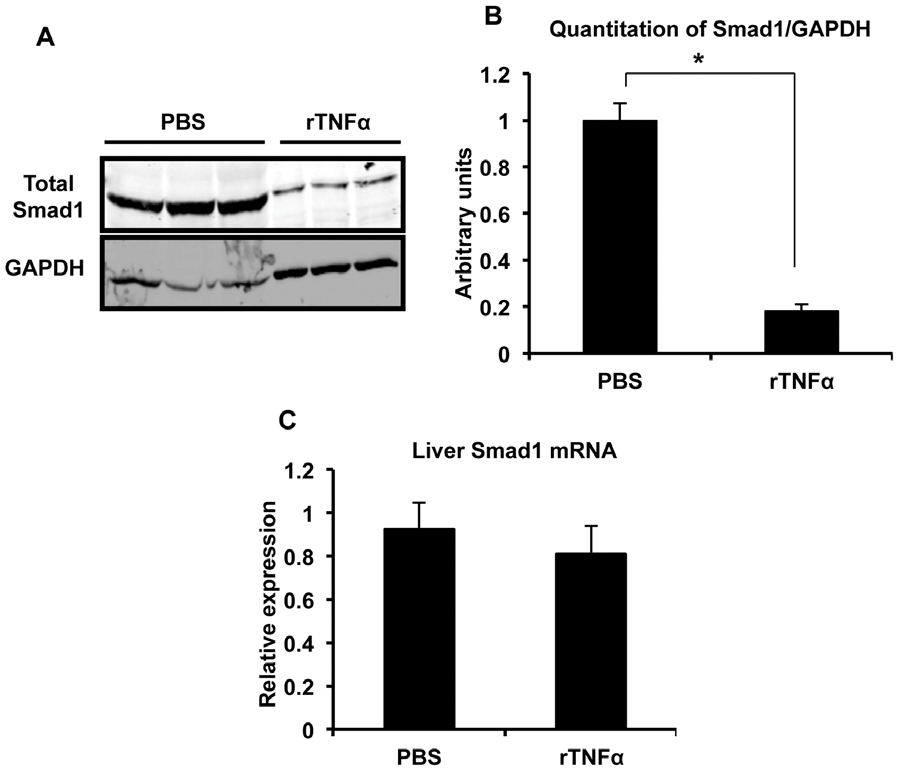
Effects of recombinant TNFα on the BMP/Smad pathway in non-colitic mice. **A**, Immunoblotting of liver lysates for total Smad1 and GAPDH in non-colitic mice treated with PBS or 50 µg/kg body weight recombinant TNFα (rTNFα) followed by sacrifice 16 hours later. Each lane represents an individual mouse. The lanes with the lysates from the rTNFα-treated mice have been over-loaded to clearly illustrate the decrease in Smad1 levels. **B**, Quantitation of band intensities from immunoblotting. *p = 0.0001, **p = 0.0004, ***p = 0.01, n = 3 mice per group. **C**, Liver Smad1 mRNA levels in the non-colitic mice treated with PBS or rTNFα, shown relative to the mean of PBS-treated mice after normalizing to GAPDH. n = 8 (control), 6 (rTNFα).

We carried out additional experiments with the DSS colitis model to further elucidate the mechanism of hepcidin down-regulation. We focused on the BMP signaling pathway given its important role in regulation of hepcidin expression [9], [20]–[22]. We found that hepatic expression of the Smad1 protein, one of the receptor-associated Smads that transduces signals from the BMP receptor complex, was decreased significantly in the mice with DSS colitis (Figure 5). Similar results were obtained with an antibody recognizing all 3 receptor-associated Smads involved in BMP signaling, Smads 1, 5 and 8 (Figure S4). Phosphorylation itself appeared to occur normally, as indicated by a trend towards an increase in the ratio of phosphorylated to total Smad (Figure 5). The DSS colitis-induced decrease in Smad1 protein expression was prevented by TNFα neutralization (Figures 6A and 6B). The anti-TNFα treatment did not significantly alter levels of the Smad1 mRNA (Figure 6C), suggesting that the effect of TNFα on Smad1 expression was at the level of translation or post-translational stability. Substantiating the results of the anti-TNFα experiments, we found that treatment of non-colitic mice with recombinant TNFα led to a reduction in Smad1 protein levels (Figures 7A and 7B) without significant effects on Smad1 mRNA (Figure 7C). Moreover, the treatment with TNFα led to a significant decrease in expression of Id1, a transcriptional target of the BMP/Smad pathway [22] (Figure S5). Taken together, our findings suggest that the inhibitory effect of TNFα on hepcidin expression during DSS colitis is mediated by down-regulation of Smad1 protein (and possibly Smad5 and Smad8) and consequent interference with BMP-activated signals.

## Discussion

There has been a great deal of interest in understanding how hepcidin is regulated in IBD because of the role played by this hormone in the pathogenesis of AI [1], [2]. Elucidation of the mechanisms that lead to hepcidin up-regulation in inflammatory states such as IBD is an essential first step in the development of strategies to inhibit this up-regulation and thereby prevent or treat AI. Earlier experiments from our laboratory have also raised the possibility that, in addition to its role in AI, hepcidin may contribute to the inflammatory process itself by increasing intra-macrophage iron and promoting the production of pro-inflammatory cytokines [8], [9]. Thus, inhibiting hepcidin expression could have the dual benefit of helping to correct AI and to ameliorate inflammation. The work described here, together with our previously published findings [9], provides important mechanistic insights into the regulation of hepcidin during intestinal inflammation.

Our earlier experiments demonstrated that hepcidin expression is increased during multiple forms of intestinal inflammation in mice, including *Salmonella* enterocolitis, the initial phase of DSS colitis, piroxicam/IL-10 knockout colitis and T cell transfer colitis, with BMPs 2, 4 and 6 playing a positive regulatory role in this process [8], [9]. IL-6 had a similar up-regulating effect in early DSS colitis [9]. Others have demonstrated the up-regulation of hepcidin in human and murine malaria, BMP- and IL-6-dependent in the latter, and a TLR2-dependent, IL-6-independent increase in *Borrelia* infection of mice [27]–[30]. Among these examples of in vivo inflammation, murine malaria (ref. 29) and DSS and TRUC colitis (the present work) appear to be unique in manifesting decreased expression of hepcidin in the later stages of the inflammatory process. Our findings have shed light on the mechanism of hepcidin down-regulation in the latter models by implicating TNFα. Although TNFα has been shown previously to inhibit hepcidin expression in a hepatocyte cell line [15], the results shown here represent, to the best of our knowledge, the first demonstration that this cytokine has a negative regulatory effect on hepcidin in clinically relevant in vivo models of inflammation. The mechanism by which TNFα inhibits hepcidin expression appears to involve decreased expression of the Smad1 protein (possibly together with Smad 5 and Smad8), an essential component of the machinery that transduces the BMP-induced signals required for hepcidin expression [22]. TNFα did not alter expression of the Smad1 mRNA, suggesting that the effect was at the level of translation or post-translational stability. The Smad1 protein has been shown to be regulated by proteasomal degradation following sequential phosphorylation of the carboxy-terminus by mitogen-activated protein kinases and glycogen synthase kinase 3 [31]. Further studies will be required to determine whether this mechanism might explain the effect of TNFα on Smad1 expression, and whether the decrease in Smad1 expression is sufficient to explain TNFα-induced down-regulation of hepcidin. TNFα has been reported to have other effects on the BMP signaling pathway, including induction of the inhibitor Smad7, repression of BMP4 and the co-receptor hemojuvelin, induction of the ubiquitin ligase Smurf1, and interference with the binding of Smad1-Smad4 complexes to their target DNA [32]–[36]. It is possible that such effects may also contribute to TNFα-induced down-regulation of hepcidin, and additional experiments will be required to clarify this issue in a definitive fashion.

Despite the clear involvement of TNFα in regulation of hepcidin expression in DSS colitis and TRUC colitis, we found that neutralization of the cytokine had no effect on hepcidin expression in the spontaneous T cell-mediated intestinal inflammation that occurs in mice with hematopoietic- and endothelial-specific deficiency of the αv integrin [37] (data not shown). TNFα is expressed in this model at levels comparable to DSS colitis, and indeed anti-TNFα treatment attenuates the severity of the colitis in the αv integrin knockout mice. Therefore, the failure of TNFα neutralization to alter hepcidin expression in these animals raises the possibility that TNFα-dependent down-regulation of hepcidin may be counteracted by other factors during T cell-mediated intestinal inflammation. Although the identity and mechanism of action of such factors awaits elucidation, this idea would be consistent with our earlier observations demonstrating increased hepcidin expression in mice with piroxicam/IL-10 knockout colitis and T cell transfer colitis [8], [9], as well as with the results of those studies of human IBD that demonstrate elevated hepcidin levels [5], [6]. Taken together, these observations suggest that the regulation of hepcidin expression may vary depending on the type of intestinal inflammation being considered, and that net hepcidin expression may be determined by whether positive or negative influences dominate. Such variations may contribute to the inconsistencies in hepcidin levels that have been noted in studies of human IBD. Detailed characterization of the factors that regulate hepcidin expression in different forms of intestinal inflammation will be required in order to develop rational strategies for the therapeutic modulation of hepcidin in IBD.

Our experiments have revealed a previously unappreciated role for TNFα in the down-regulation of hepcidin expression in at least some forms of intestinal inflammation. This function may be important in helping to maintain serum iron levels in the face of inflammatory insults. It is also possible that TNFα-induced down-regulation of hepcidin may have evolved as a mechanism to protect against intracellular pathogens. Several studies have shown that hepcidin-dependent changes in the levels of ferroportin expressed by macrophages have a significant influence on the growth of pathogens that reside in these cells, such as *Salmonella*, *Chlamydia* and *Mycobacteria* [38]–[40]. By decreasing the amount of hepcidin produced by the liver, TNFα would allow ferroportin expression on macrophages to go up, thereby increasing iron efflux from these cells and depriving any intracellular pathogens of this essential nutrient. Given the importance of TNFα in IBD pathogenesis, the increasing use of anti-TNFα therapy to control the disease, and the risk of tuberculosis associated with such therapy [41], it will be important to determine if TNFα has inhibitory effects on hepcidin expression in human IBD.

## Supporting Information

Figure S1 **TNFα expression during DSS colitis.** **A,** TNFα secreted by colon explants from control, untreated mice (Con), or mice treated for 3 or 7 days with DSS. *p = 0.03, **p = 0.005, n = 10 for control group, 9 for each of the DSS-treated groups. **B,** IL-6 secreted by colon explants from control, untreated mice (Con), or mice treated for 3 or 7 days with DSS. *p = 0.0001, n = 10 for control and 3-day DSS treated groups, 9 for the 7-day DSS treated group.
(PDF)

Figure S2 **Effect of TNFα on lactate dehydrogenase (LDH) release in Huh7 cells.** Huh7 cells were treated with 10 ng/ml of TNFα for 12 hours. LDH released into the supernatant was measured using the CytoTox96 kit (Promega, Madison, WI) and expressed as a percentage of the amount in the cell lysate.
(PDF)

Figure S3 **Effects of TNFα neutralization on DSS colitis.** Colon length (**A**) and colon IL-6 secretion (**B**) were measured following 7 days of DSS treatment during which the mice were injected with PBS or anti-TNFα. In **A**, * p = 0.001, n = 5 (control), 10 (anti-TNFα). In **B**, *p = 0.05, n = 5 (control), 10 (anti-TNFα).
(PDF)

Figure S4 **Alterations in Smad1/5/8 expression during DSS colitis** **A**, Immunoblotting of liver lysates for total Smad1/5/8 and actin in control, untreated mice (Con), or mice treated for 3 or 7 days with DSS. Each lane represents an individual animal. **B**, Quantitation of band intensities from the immunoblotting experiment. *p = 0.047, n = 3 in each group.
(PDF)

Figure S5 **Effect of TNFα on Id1 expression in vivo.** Liver Id1 mRNA levels in control (Con) mice, or mice treated with recombinant TNFα (rTNFα) 50 µg/kg body weight followed by sacrifice 16 hours later. Id1 expression is shown relative to the mean of the controls after normalizing to GAPDH. *p = 0.011, n = 5 in each group.
(PDF)
