# Supplementary figures and images for: Tumor Necrosis Factor α Inhibits Expression of the Iron Regulating Hormone Hepcidin in Murine Models of Innate Colitis
Source: PLoS One. 2012 May 31;7(5):e38136. doi: 10.1371/journal.pone.0038136 (PMC3365004; doi:10.1371/journal.pone.0038136)

Figure S1

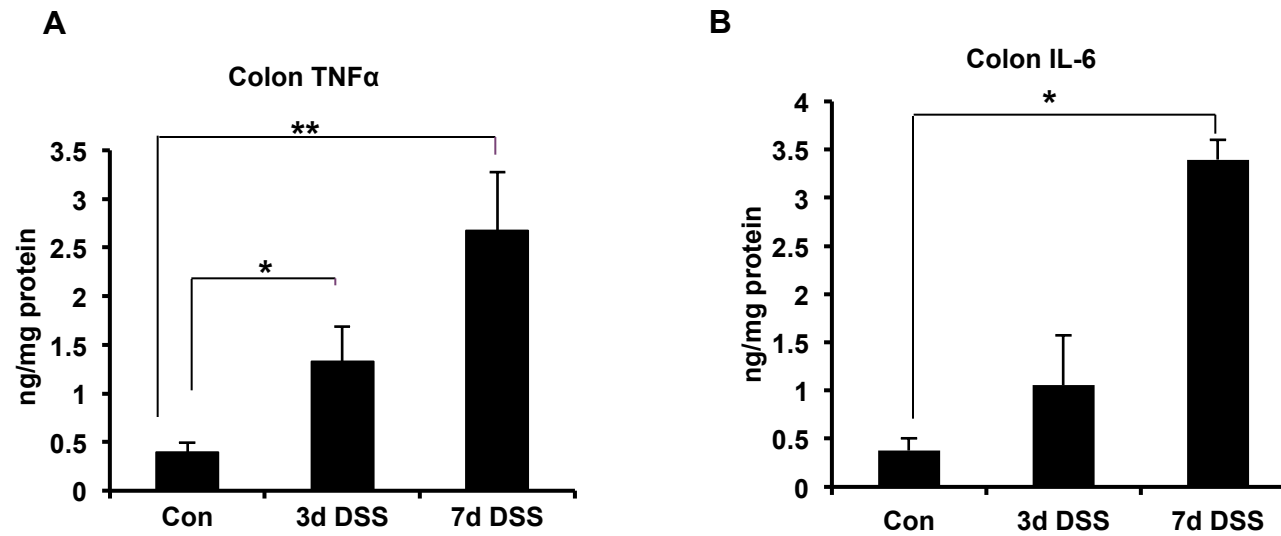

Supplement: Figure S1 — TNFα expression during DSS colitis. A, TNFα secreted by colon explants from control, untreated mice (Con), or mice treated for 3 or 7 days with DSS. *p = 0.03, **p = 0.005, n = 10 for control group, 9 for each of the DSS-treated groups. B, IL-6 secreted by colon explants from control, untreated mice (Con), or mice treated for 3 or 7 days with DSS. *p = 0.0001, n = 10 for control and 3-day DSS treated groups, 9 for the 7-day DSS treated group. (PDF) [file pone.0038136.s001.pdf]

**Figure S2**

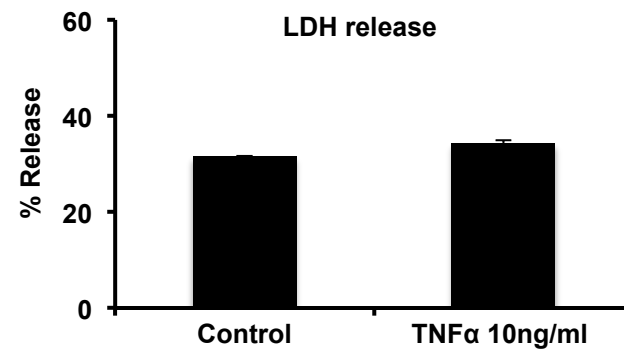

Supplement: Figure S2 — Effect of TNFα on lactate dehydrogenase (LDH) release in Huh7 cells. Huh7 cells were treated with 10 ng/ml of TNFα for 12 hours. LDH released into the supernatant was measured using the CytoTox96 kit (Promega, Madison, WI) and expressed as a percentage of the amount in the cell lysate. (PDF) [file pone.0038136.s002.pdf]

Figure S3

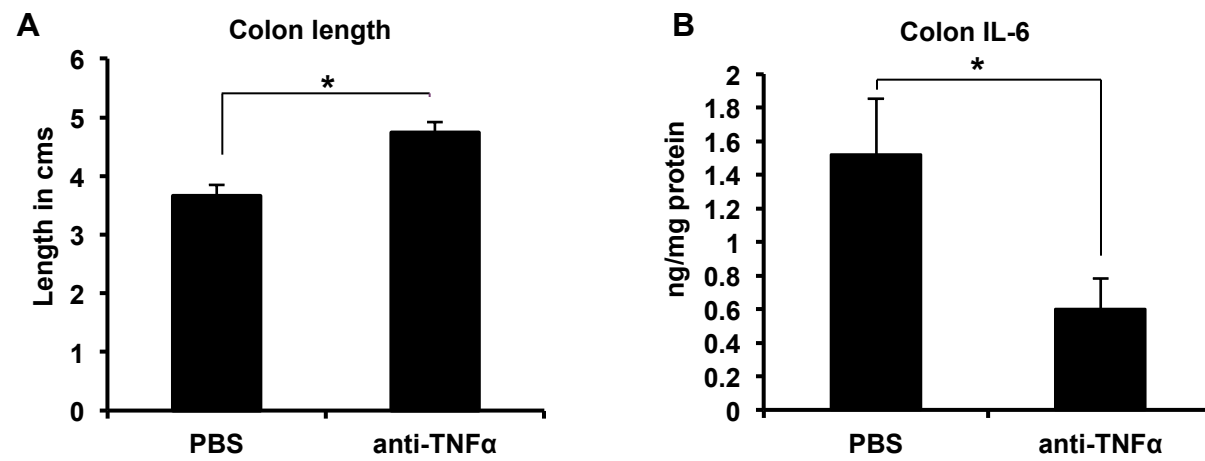

Supplement: Figure S3 — Effects of TNFα neutralization on DSS colitis. Colon length (A) and colon IL-6 secretion (B) were measured following 7 days of DSS treatment during which the mice were injected with PBS or anti-TNFα. In A, * p = 0.001, n = 5 (control), 10 (anti-TNFα). In B, *p = 0.05, n = 5 (control), 10 (anti-TNFα). (PDF) [file pone.0038136.s003.pdf]

**Figure S4**

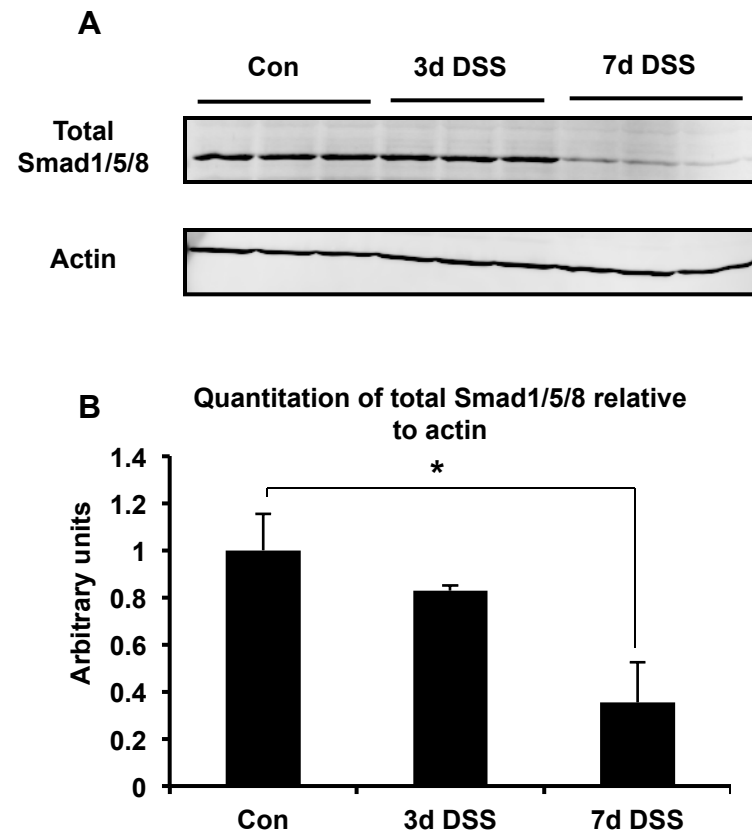

Supplement: Figure S4 — Alterations in Smad1/5/8 expression during DSS colitis A, Immunoblotting of liver lysates for total Smad1/5/8 and actin in control, untreated mice (Con), or mice treated for 3 or 7 days with DSS. Each lane represents an individual animal. B, Quantitation of band intensities from the immunoblotting experiment. *p = 0.047, n = 3 in each group. (PDF) [file pone.0038136.s004.pdf]

Figure S5

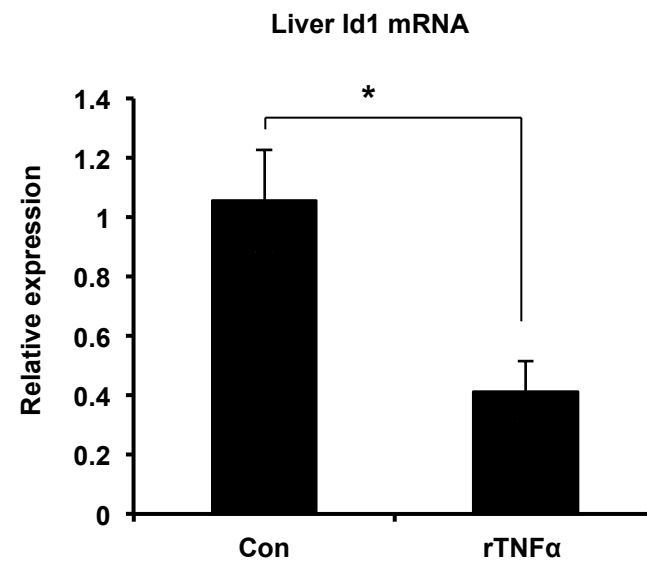

Supplement: Figure S5 — Effect of TNFα on Id1 expression in vivo. Liver Id1 mRNA levels in control (Con) mice, or mice treated with recombinant TNFα (rTNFα) 50 µg/kg body weight followed by sacrifice 16 hours later. Id1 expression is shown relative to the mean of the controls after normalizing to GAPDH. *p = 0.011, n = 5 in each group. (PDF) [file pone.0038136.s005.pdf]
